# COVID-19-Impfstrategien für Beschäftigte deutscher Kliniken: Ergebnisse einer Befragung von Leitungen der (Krankenhaus‑)Hygiene im Rahmen des B-FAST-Projektes

**DOI:** 10.1007/s00103-022-03607-x

**Published:** 2022-10-21

**Authors:** Simone Scheithauer, Anna Bludau, Stephanie Heinemann, Martina Anton, Percy Knolle

**Affiliations:** 1 Institut für Krankenhaushygiene und Infektiologie, Universitätsmedizin Göttingen, Georg-August-Universität Göttingen, Robert-Koch-Straße 40, 37075 Göttingen, Deutschland; 2 Lokale Task Force Netzwerk Universitätsmedizin (NUM), Universitätsmedizin Göttingen, Georg-August-Universität Göttingen, Göttingen, Deutschland; 3 Institut für Allgemeinmedizin, Universitätsmedizin Göttingen, Georg-August-Universität Göttingen, Göttingen, Deutschland; 4 Institut für Molekulare Immunologie und Experimentelle Onkologie, Fakultät für Medizin, Universitätsklinikum rechts der Isar der Technischen Universität München, München, Deutschland; 5 Standort München, Deutsches Zentrum für Infektionsforschung, München, Deutschland; 6 Lokale Task Force Netzwerk Universitätsmedizin (NUM), Fakultät für Medizin, Technische Universität München, München, Deutschland

**Keywords:** SARS-CoV‑2, Impfung, Impfkampagne, Antikörper-Testung, Durchbruchsinfektion, SARS-CoV‑2, Vaccination, Immunization programs, Antibodies, COVID-19 testing

## Abstract

**Hintergrund und Ziel:**

Zu Beginn der COVID-19-Impfkampagne in Deutschland wurden Beschäftigte in medizinischen Einrichtungen aufgrund des hohen Expositionsrisikos und des Kontakts mit vulnerablen Gruppen priorisiert gegen SARS-CoV‑2 geimpft. Die Krankenhäuser waren angehalten die Impfungen ihrer Beschäftigten möglichst schnell zu organisieren und durchzuführen. Ziel der vorliegenden Arbeit war es, die Impfstrategie für die Mitarbeitenden deutscher Kliniken zu erfassen.

**Methoden:**

In einer Querschnittstudie mit selbstentwickeltem Fragebogen wurden die Leitungen der (Krankenhaus‑)Hygiene aller deutschen Universitätskliniken sowie der Nicht-Universitätskliniken in Niedersachsen und Bayern im März 2021 befragt. Die Daten wurden nach den beiden Versorgungsstufen stratifiziert.

**Ergebnisse:**

100 von 416 versendeten Fragebögen wurden vollständig ausgefüllt (Universitätsklinik: 33, Nicht-Universitätsklinik: 67). Universitätskliniken berichteten von einer größeren Impfkapazität als Nicht-Universitätskliniken, ein begrenzender Faktor waren die ungewissen Impfstofflieferungen. 89 % der Kliniken planten Informationskampagnen zum Thema Impfung oder hatten diese bereits durchgeführt. 70 % gaben an, keine Antikörpertests bei geimpften Beschäftigten durchführen zu wollen. Eine Nachverfolgung geimpfter Beschäftigter zur Detektion möglicher SARS-CoV-2-Infektionen mittels Erregernachweis durch PCR wurde von 41 % geplant. Im Falle des Nachweises einer SARS-CoV-2-Infektion bei geimpften Beschäftigten hatten 72 % weitere Diagnostik geplant.

**Diskussion:**

Alle Krankenhäuser konnten eine schnelle Umsetzung der COVID-19-Vakzinierung ihrer Beschäftigten erreichen. Zum Zeitpunkt der Befragung gab es große Unsicherheit bezüglich des Umgangs mit Durchbruchsinfektionen und der Notwendigkeit von Auffrischimpfungen.

**Zusatzmaterial online:**

Zusätzliche Informationen sind in der Online-Version dieses Artikels (10.1007/s00103-022-03607-x) enthalten.

## Abstract (en)

**Background and aim:**

At the beginning of the COVID-19 vaccination campaign in Germany, employees in medical facilities were prioritised for vaccination against SARS-CoV‑2 due to the high risk of exposure and contact with vulnerable groups. Hospitals were therefore encouraged to organise and implement the vaccination of their employees as soon as possible. The aim of the study was to record the practice regarding the vaccination strategy for employees in German hospitals.

**Methods:**

In a self-developed cross-sectional study, infection control practitioners of all German university hospitals as well as non-university hospitals in Lower Saxony and Bavaria were surveyed in March 2021. The data were stratified according to the characteristics of university hospitals and non-university hospitals.

**Results:**

Of 416 invitations sent out, 100 questionnaires (university hospitals: 33; non-university hospitals: 67) were completed. University hospitals reported greater vaccination capacity than non-university hospitals, but a limiting factor was uncertain vaccine supply. Vaccination information campaigns were planned or had already been conducted in 89% of clinics. About two-thirds of the respondents (70%) said they did not plan to conduct antibody tests on vaccinated employees. A follow-up of vaccinated employees to detect possible SARS-CoV‑2 infections by PCR was planned by 41% of the respondents. In case of detection of SARS-CoV‑2 infection, 72% of the respondents had planned further diagnostic procedures.

**Discussion:**

All hospitals were able to achieve rapid implementation of COVID-19 vaccination of their employees. At the time of the survey, there was also much uncertainty regarding the management of breakthrough infections as well as the need for booster vaccinations.

## Hintergrund

Während der SARS-CoV-2-Pandemie haben deutsche Kliniken unter hohem Zeitdruck neue Strategien im Bereich Infektionsprävention zum Schutz von Patient*innen und Beschäftigten implementieren müssen. Einen wichtigen Faktor stellt in diesem Kontext die COVID-19-Impfung dar. Zu Anfang der Impfkampagne in Deutschland, die im Dezember 2020 startete, waren die Impfstoffe nur begrenzt verfügbar [1]. Das Personal in medizinischen Einrichtungen wurde aufgrund des hohen Expositionsrisikos sowie des Kontakts mit vulnerablen Gruppen priorisiert geimpft [2]. Kliniken waren daher angehalten die Impfungen ihrer Beschäftigten möglichst schnell zu organisieren und durchzuführen.

Seit Ende 2020 standen in Deutschland mRNA-basierte Impfstoffe von BioNTech/Pfizer und Moderna sowie Vektor-basierte Impfstoffe von AstraZeneca für die Impfung zur Verfügung [3]. Eine Studie des Robert Koch-Instituts (RKI) vom 22.03.2021 bis 12.04.2021 zeigte, dass 83 % des teilnehmenden Krankenhauspersonals zu dem Zeitpunkt bereits mindestens eine Impfdosis erhalten hatten [3]. Jedoch war ein nicht vorhandenes Impfangebot der Hauptgrund für eine fehlende Impfung [3]. Ein weiterer wichtiger Aspekt war die Durchführung von gezielten und gut strukturierten Informationskampagnen, da sich das Krankenhauspersonal auf der einen Seite nicht ausreichend informiert fühlte [3], es auf der anderen Seite aber auch einen Überfluss an Informationen und Falschinformationen gab [4–6]. Dies trug offenbar zur Unsicherheit gegenüber einer Impfung bei, welche zu Unentschlossenheit oder Ablehnung geführt hatte [3]. Auch Themenfelder wie Knappheit an Impfdosen, Zweitimpfungen, die Notwendigkeit von Antikörpertests zur Überprüfung der Effizienz der Impfung [7, 8] sowie der Umgang mit möglichen Durchbruchsinfektionen bei vollständig Geimpften [9–13] waren zu der Zeit in Diskussion.

Um die Praxis bezüglich der COVID-19-Impfung, die Vorbereitung auf die Wintersaison 2021 sowie die Teststrategie bei geimpften Beschäftigten in deutschen Kliniken zu erfassen, wurde im Rahmen des „Bundesweiten Forschungsnetzwerkes zu Angewandter Surveillance und Testung“ (B-FAST) im Netzwerk Universitätsmedizin (NUM) im März 2021 eine standardisierte Befragung durchgeführt. Es wurden alle deutschen Universitätskliniken sowie die Nicht-Universitätskliniken in Niedersachsen und Bayern ausgewählt, um ein möglichst repräsentatives Bild zu erhalten. Die Unterschiede zwischen Universitätskliniken und Nicht-Universitätskliniken wurden untersucht, da von verschiedenen personellen, materiellen und infrastrukturellen Ressourcen dieser Einrichtungen sowie deren Einfluss auf Organisation und Durchführung von Impfungen bei Beschäftigten ausgegangen wurde.

Folgende Fragen wurden adressiert:

1. In welchem Zeitraum und mit welcher täglichen Impfkapazität werden die Impfungen von Beschäftigten in deutschen Kliniken durchgeführt?
2. Werden Informationskampagnen zur COVID-19-Impfung durchgeführt? Welcher Inhalt wird darin behandelt?
3. Sind Antikörpertests bei geimpften Beschäftigten zur Überprüfung der Effizienz der Vakzinierung geplant?
4. Wird eine Drittimpfung für vollständig geimpfte Beschäftigte vor der Wintersaison 2021 in Betracht gezogen?
5. Welche Strategie zur Detektion von Durchbruchsinfektionen mit SARS-CoV‑2 bei geimpften Beschäftigten wird verfolgt?

## Methoden

### Studiendesign und Teilnehmende

In einer Querschnittstudie mit einem selbstentwickelten Fragebogen wurden Leitungen der (Krankenhaus‑)Hygiene deutscher Kliniken zu verschiedenen Aspekten ihrer SARS-CoV-2-Test- und Surveillance-Strategie befragt. Ein Fokus lag auf der COVID-19-Impfung von Beschäftigten und soll in dieser Ausarbeitung näher betrachtet werden.

Der Fragebogen richtete sich an folgende Stichprobe: 1) alle deutschen Universitätskliniken (*n* = 36), 2) Nicht-Universitätskliniken in Niedersachsen (*n* = 115) und 3) Nicht-Universitätskliniken in Bayern (*n* = 265). Nicht-Universitätskliniken umfassen alle Akutkrankenhäuser ohne Universitätsstatus unabhängig von Versorgungsgrad und Trägerschaft. Die Bundesländer wurden aufgrund ihrer unterschiedlich hohen Inzidenzwerte in dem der Befragung vorangegangenen Jahr 2020 ausgewählt. Befragungseinheit waren keine einzelnen Personen, sondern die Organisationen. Eine ausführliche Beschreibung der Fragebogenentwicklung sowie Strategien zur Zielgruppenerreichung können im Onlinematerial 1 zu diesem Beitrag nachgelesen werden.

Der Fragebogen wurde in einem interdisziplinären Verfahren mit Vertreter*innen aus den Bereichen Virologie, Immunologie, Infektionsmedizin, Hygiene und Public Health entworfen und getestet. Die Befragung wurde zwischen dem 01. und 25.03.2021 durchgeführt. Die Auswertung erfolgte zusätzlich mit einem oder einer Vertreter*in der Arbeitsmedizin.

Das Vorgehen wurde final von dem Datenschutzbeauftragten am 08.02.2021 (B-FAST/tl) und der Ethikkommission der Universitätsmedizin Göttingen (UMG) am 29.01.2021 (5/2/25) bewilligt.

### Statistische Analyse

Zur Datenauswertung wurde IBM SPSS Statistics 26 genutzt. Die Daten wurden nach den Merkmalen „Universitätsklinik“ und „Nicht-Universitätsklinik“ stratifiziert. Zur Prüfung der statistischen Signifikanz der Unterschiede zwischen Universitätsklinik und Nicht-Universitätsklinik wurde der exakte Test nach Fisher verwendet. Bei Multiple-Choice-Fragen wurde der Test pro Item durchgeführt. Bei Single-Choice-Fragen wurde der Test für die gesamte Frage durchgeführt. Wenn das Ergebnis signifikant war, wurden exakte Tests nach Fisher für die Post-hoc-Analyse verwendet und mithilfe der Bonferroni-Methode adjustiert. Statistische Signifikanz wurde, wenn nicht anders indiziert, als *p* < 0,05 definiert.

## Ergebnisse

Von 416 versendeten Einladungen zur Befragung wurden 100 Fragebögen vollständig ausgefüllt. Bei den Universitätskliniken konnte ein Rücklauf von 91,6 % (33 von 36) erzielt werden. Bei den Nicht-Universitätskliniken wurde ein Rücklauf von 32,2 % (37 von 115) in Niedersachsen und von 11,3 % (30 von 265) in Bayern erreicht. 87,9 % der Universitätskliniken hatten über 1000 Betten, während dies nur auf 3,0 % der Nicht-Universitätskliniken zutraf. Weitere Charakteristika der Kliniken bezüglich ihrer personellen, materiellen und infrastrukturellen Ressourcen sind im Onlinematerial 2 dargestellt und wurden auch bereits an anderer Stelle bezüglich der Test- und Surveillance-Strategien berichtet [14].

Nachfolgend werden die Forschungsfragen 1 bis 5 einzeln aufgegriffen und deren Ergebnisse bezogen auf die gesamte Stichprobe und nach Versorgungsstufe berichtet.

1. **Zeitraum der COVID-19-Vakzinierungen**.

**Figure 1:**
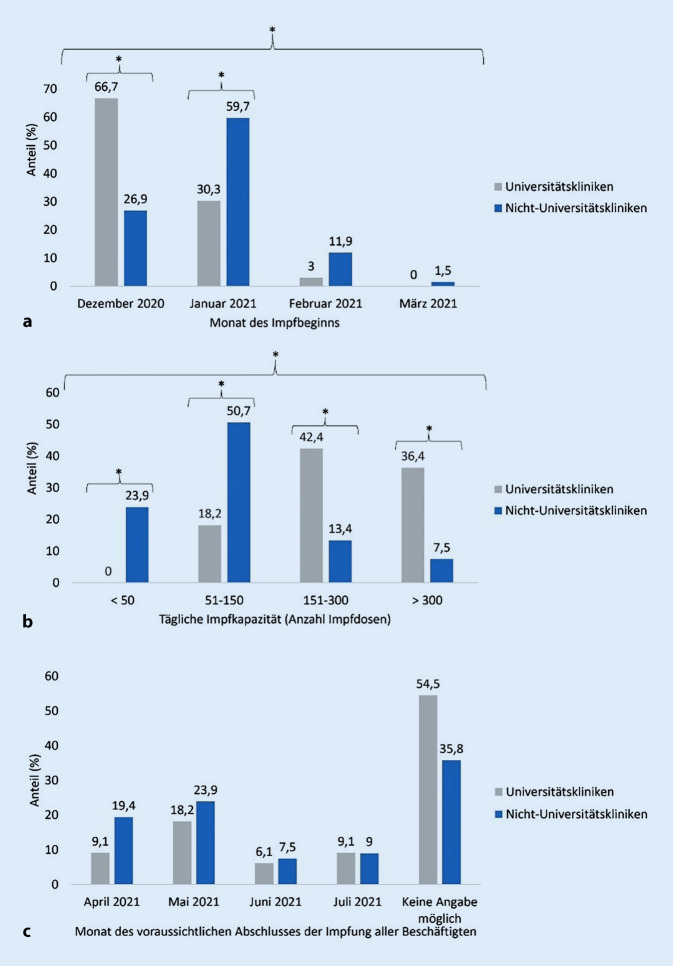

Alle Krankenhäuser hatten zum Zeitpunkt der Befragung bereits mit der Impfung ihrer Beschäftigten angefangen. Begonnen hatten die Impfungen in 40,0 % aller befragten Krankenhäuser bereits im Dezember 2020; ca. 50,0 % der Krankenhäuser begannen im Januar 2021. 2 Drittel der Universitätskliniken (UK), signifikant mehr als Nicht-Universitätskliniken (NUK), hatten bereits im Dezember mit den Impfungen begonnen (UK: 66,7 %, NUK: 26,9 %, *p* = 0,0002; Abb. 1a). Über die Hälfte der Nicht-Universitätskliniken starteten die Impfungen dagegen erst im Januar (UK: 30,3 %, NUK: 59,7 %, *p* = 0,0102). Nur eine geringe Anzahl der Einrichtungen berichteten einen späteren Impfstart im Februar (9,0 %, *p* = 0,2646) oder März (1,0 %, *p* = 1,0) 2021.

Bei der Frage nach der täglichen Impfkapazität konnte zwischen 4 Kategorien gewählt werden. 17,0 % der Krankenhäuser gaben an, täglich über 300 Impfungen durchführen zu können (UK: 36,4 %/12, NUK: 7,5 %/5, *p* = 0,0011; Abb. 1b). Zwischen 151 und 300 Impfungen pro Tag konnten 23,0 % der befragten Einrichtungen leisten (UK: 42,4 %/14, NUK: 13,4 %/9, *p* = 0,0022). Befragte in 40,0 % aller Einrichtungen gaben an, dass sie täglich zwischen 51 und 150 Beschäftigte impfen können (UK: 18,2 %/6, NUK: 50,7 %/34, *p* = 0,0019). Einige der Nicht-Universitätskliniken gaben an, dass sie bei ausreichend Impfstoff täglich bis zu 50 Impfungen durchführen können (UK: 0 %/0, NUK: 23,9 %/16, *p* = 0,0010).

Zum Befragungszeitpunkt gingen 16,0 % der Krankenhäuser davon aus, dass alle Beschäftigten im April 2021 geimpft sein werden. In 22,0 % der teilnehmenden Krankenhäuser wurde geschätzt, dass die Impfungen im Mai 2021 abgeschlossen sein werden. Fast jede zweite Einrichtung (42,0 %) konnte allerdings keine konkreten Angaben zum Zeitpunkt der abgeschlossenen Impfungen machen, da Impfstofflieferungen zum Befragungszeitpunkt im März 2021 ungewiss waren. Hier ergaben sich keine signifikanten Unterschiede zwischen Universitätskliniken und Nicht-Universitätskliniken (Abb. 1c).

2. **Informationskampagnen zur Impfung**.

Insgesamt planten 89,0 % der Häuser zum Befragungszeitpunkt Informationskampagnen zum Thema Impfung oder hatten diese bereits durchgeführt (Tab. 1). Anhand dieser Kampagnen sollten Beschäftigte vor allem über den Impfschutz (86,0 %) und mögliche Begleitreaktionen von der COVID-19-Vakzinierung (83,0 %) aufgeklärt werden. Universitätskliniken schlossen signifikant häufiger den Ablauf der Impfanmeldung in Ihre Informationen mit ein (UK 90,9 %, NUK 64,2 %; *p* = 0,043).

**Table 1:** 

	Universitätskliniken (*n* = 33)		Nicht-Universitätskliniken (*n* = 67)		Gesamt (*n* = 100)	*p*-Wert
	Relativ	Absolut	Relativ	Absolut		
**Durchführung von Informationskampagnen**	97,0	32	76,1	51	89,0^a^	0,084
**Inhalte der Informationskampagnen**						
Impfschutz	97,0	32	80,6	54	86,0^a^	0,550
Mögliche Nebenwirkungen/Begleitreaktionen	97,0	32	76,1	51	83,0^a^	0,084
Aufbau und Wirkungsweise der SARS-CoV-2-Impfstoffe	87,9	29	71,6	51	77,0^a^	0,525
Ablauf der Anmeldung zur Impfung	90,9	30	64,2	43	73,0^a^	**0,043**
Ablauf der Impfung	78,8	26	64,2	43	69,0^a^	0,605
Priorisierung	78,8	26	62,7	42	68,0^a^	0,604
Verfügbarkeit von Impfstoffen	72,7	24	53,7	36	60,0^a^	0,347

^a^ Absolute Zahlen sind gleich den relativen Zahlen

3. **Bestimmung von SARS-CoV-2-Antikörpern**.

Knapp über 2 Drittel der Befragten (70,0 %) gaben an, keine systematischen Antikörpertests bei geimpften Beschäftigten durchführen zu wollen (Tab. 2). Unsicherheit oder Unentschlossenheit hinsichtlich einer Bestimmung von SARS-CoV-2-spezifischen Antikörpern äußerten 12,0 %, dagegen hatten 18,0 % einen fest definierten Zeitpunkt für die Bestimmung der Antikörper in ihrer Institution vorgesehen. Hinsichtlich dieser Ergebnisse unterschieden sich Universitätskliniken und Nicht-Universitätskliniken nicht signifikant voneinander. 72,2 % der befragten Institutionen mit einem fest definierten Zeitpunkt für Bestimmung der Antikörper planten eine Bestimmung von Anti-SARS-CoV-2-IgG (alle Antikörper) und 33,3 % gezielt eine Bestimmung der neutralisierenden Antikörper gegen SARS-CoV‑2 als Surrogat für den Impferfolg (Mehrfachantworten waren möglich).

**Table 2:** 

	Universitätskliniken (*n* = 33)		Nicht-Universitätskliniken (*n* = 67)		Gesamt (*n* = 100)		*p*-Wert
	Relativ	Absolut	Relativ	Absolut	Relativ	Absolut	
**Zeitpunkt der SARS-CoV-2-spezifischen Antikörperbestimmung bei geimpften Beschäftigten**	–	–	–	–	–	–	0,868
Zu einem definierten Zeitpunkt nach der zweiten Impfung	9,1	3	7,5	5	8,0	8	–
Vor Beginn der Wintersaison	3,0	1	4,5	3	4,0	4	–
Anderer Zeitpunkt/innerhalb von Studien	18,2	6	0,0	0	6,0	6	–
Noch nicht bekannt/noch nicht entschieden	9,1	3	13,4	9	12,0	12	–
**Keine Antikörpertests bei geimpften Beschäftigten geplant**	60,6	20	74,6	50	70,0	70	–
**Form des Nachweises** (Mehrfachauswahl möglich)	(*n* = 10)		(*n* = 8)		(*n* = 18)		–
Bestimmung von Anti-SARS-CoV-2-IgG (alle Antikörper)	70,0	7	75,0	6	72,2	13	0,460
Bestimmung von neutralisierenden Antikörpern gegen SARS-CoV‑2	30,0	3	37,5	3	33,3	6	0,460

4. **COVID-19-Drittimpfung**.

In einem Großteil der befragten Krankenhäuser (80,0 %) war noch ungeklärt, ob eine dritte COVID-19-Vakzinierung als „Booster-Impfung“ für Beschäftigte vor Beginn der Wintersaison 2021 in Betracht gezogen werden sollte (Tab. 3). Es gab hier keine signifikanten Unterschiede zwischen den Versorgungsstufen. Die Entscheidung für eine Drittimpfung für Beschäftigte wurde zu diesem Zeitpunkt von 77,0 % der Kliniken von den verfügbaren Informationen über die Wirksamkeit von COVID-19-Vakzinen abhängig gemacht und von 75,0 % der Kliniken von Informationen über die zirkulierenden Virusvarianten (Variants of Concern – VoC) mit Potenzial für „Immunescape“ (Immunflucht). Eine Mehrfachauswahl war hier möglich.

**Table 3:** 

	Universitätskliniken (*n* = 33)		Nicht-Universitätskliniken (*n* = 67)		Gesamt (*n* = 100)	*p*-Wert
	Relativ	Absolut	Relativ	Absolut		
**Mögliche Durchführung einer Booster-Impfung**	–	–	–	–	–	0,888
Ja	15,2	5	16,4	11	16,0^a^	–
Nein	3,0	1	4,5	3	4,0^a^	–
Noch unbekannt	81,8	27	79,1	53	80,0^a^	–
**Relevante Informationen für die Entscheidung** (Mehrfachauswahl möglich)						
Informationen über Wirksamkeit von COVID-19-Vakzinen	87,9	29	71,6	48	77,0^a^	0,102
Informationen über zirkulierende Virusvarianten mit Potenzial für „Immunescape“ (Immunflucht)	81,8	27	71,6	48	75,0^a^	0,433
Informationen zur Verfügbarkeit von Impfstoffen	60,6	20	61,2	41	61,0^a^	1,0
Informationen über Antikörper-Status der Beschäftigten	21,2	7	32,8	22	29,0^a^	0,245

^a^ Absolute Zahlen sind gleich den relativen Zahlen

5. **Nachverfolgung von SARS-CoV‑2 Infektionen bei geimpften Beschäftigten**.

Eine Nachverfolgung geimpfter Beschäftigter zur Detektion möglicher SARS-CoV-2-Infektionen mittels Erregernachweis durch die Polymerase-Kettenreaktion (PCR) wurde zum Befragungszeitpunkt von 41,0 % der Befragten geplant.

Zur Antigen- oder PCR-basierten Detektion von SARS-CoV-2-Infektionen bei geimpften Beschäftigten wurde vor allem anlassbezogen (Symptome 88,0 %; Exposition 78,0 %) getestet (Tab. 4).

**Table 4:** 

	Universitätskliniken (*n* = 33)		Nicht-Universitätskliniken (*n* = 67)		Gesamt (*n* = 100)	*p*-Wert
	Relativ	Absolut	Relativ	Absolut		
**Strategie zur Detektion von SARS-CoV-2-Infektionen bei geimpften Beschäftigten** (Mehrfachauswahl möglich)						
Symptombezogene Testung	93,9	31	85,1	57	88,0^a^	0,327
Ausbruchsbezogene Testung (Exposition/Kontakt)	84,8	28	74,6	50	78,0^a^	0,310
Regelmäßige Testung bei Einsatz im COVID-19-Bereich	45,5	15	53,7	36	51,0^a^	0,525
Regelmäßige Testung unabhängig vom Einsatzbereich	30,3	10	52,2	35	45,0^a^	0,054
Testung im Rahmen einer klinischen Studie	24,2	8	1,5	1	9,0^a^	**0,001**
**Weitere Diagnostik im Falle eines positiven SARS-CoV-2-Nachweises** (Mehrfachauswahl möglich)						
Ja, Virussequenzierung	69,7	23	56,7	38	61,0^a^	0,378
Ja, Bestimmung von Antikörpern gegen SARS-CoV‑2	66,7	22	35,8	24	46,0^a^	**0,010**
Nein	18,2	6	32,8	22	28,0^a^	0,155

^a^ Absolute Zahlen sind gleich den relativen Zahlen

Im Falle eines Nachweises einer SARS-CoV-2-Infektion bei geimpften Beschäftigten hatten 72,0 % der Befragten eine weitere Diagnostik geplant. Bei 61,0 % beinhaltete dies eine Virus-Sequenzierung, bei 46,0 % die Bestimmung von Antikörpern gegen SARS-CoV‑2 (Tab. 4). Letzteres wurde signifikant häufiger in Universitätskliniken berichtet (UK: 66,7 %, NUK: 35,8 %, *p* = 0,010).

## Diskussion

Die im Frühjahr 2021 durchgeführte Umfrage bei Leitungen der (Krankenhaus‑)Hygiene zeigte, dass ein Großteil der Krankenhäuser die Impfungen bereits ab Dezember 2020 oder Januar 2021 durchführen konnte. Der begrenzende Faktor zur Bestimmung des Abschlusses der Impfungen aller Beschäftigten waren die ungewissen Impfstofflieferungen. Die meisten Krankenhäuser planten eine Informationsveranstaltung zur Impfung oder hatten diese bereits durchgeführt. Systematische Antikörpertests bei geimpften Beschäftigten wurden selten geplant, Unsicherheit herrschte auch bei der Notwendigkeit einer Booster-Impfung. Die Teststrategie bei geimpften Beschäftigten basierte eher auf anlassbezogenen Tests.

Fast alle Krankenhäuser berichteten von einer schnellen Umsetzung der COVID-19-Vakzinierung und begleitender Informationskampagnen für Beschäftigte in ihrer Institution. Die COVID-19-Vakzinierung begann in einem Großteil der Universitätskliniken rund einen Monat früher als in den meisten Nicht-Universitätskliniken. Dies lässt auf größere personelle, materielle und infrastrukturelle Ressourcen für die Organisation und Durchführung in Universitätskliniken schließen. Diese hatten ebenfalls eine höhere tägliche Impfkapazität. Interessant ist, dass in den Krankenhäusern der limitierende Faktor für die schnellstmögliche Versorgung aller Beschäftigten mit einer COVID-19-Vakzinierung eher in einer nichtausreichenden Versorgung mit Impfstoff als in strukturellen Hindernissen bei der Durchführung der Impfungen gesehen wurde. Es ist deshalb wahrscheinlich, dass zukünftige Impfempfehlungen für Beschäftigte in Krankenhäusern schnell umgesetzt werden können, solange genügend Impfstoff zur Verfügung steht.

Die im Winter bzw. Frühjahr 2021 von einem großen Teil der Krankenhäuser durchgeführte gezielte und umfassende Informationskampagne zur Impfung für Beschäftigte lässt darauf schließen, dass den Beschäftigten ausreichend Informationen zur COVID-19-Impfung zur Verfügung standen. Dies ist besonders wichtig vor dem Hintergrund, dass fehlende Informationen ein Grund für die Entscheidung gegen eine COVID-19-Vakzinierung sein können [3]. Auch wenn bislang nur wenige konkrete Zahlen zur Impfquote bei Beschäftigten im Krankenhaus vorliegen, zeigt eine rund ein Jahr später durchgeführte Umfrage des Deutschen Krankenhausinstituts (DKI), dass die Impfquote im Januar 2022 in patientennahen Bereichen bei 89 % [15] und damit deutlich über dem Durchschnitt der Impfquote in der Bevölkerung lag. Die von den Krankenhäusern betriebene Intensivierung der Informationskampagnen ist deshalb als ein wichtiges Instrument für eine ausreichende Impfung des Personals und damit auch für die Aufrechterhaltung der medizinischen Versorgung der Bevölkerung zu sehen. Mit Blick auf die Flut an Informationen und auch an Fehlinformationen aus anderen Quellen [4–6] ist es wichtig, im Austausch mit dem Beschäftigten zu bleiben und deren Feedback zur Weiterentwicklung der professionellen Kommunikationsstrategie zu nutzen.

Die vorliegende Befragung zeigte außerdem, dass SARS-CoV-2-Testungen bei geimpften Beschäftigten vor allem anlassbezogen erfolgten, also z. B. bei Vorhandensein von Symptomen oder nach Exposition, dies aber nicht von allen Kliniken so geplant war. Strategien für regelmäßige Testungen auf SARS-CoV-2-Infektionen waren zum Befragungszeitpunkt sehr heterogen. Eine nationale Teststrategie für geimpfte Personen lag noch nicht vor. Eine weitere Diagnostik im Falle eines positiven SARS-CoV-2-Nachweises war damals in 3 Viertel der Kliniken geplant. Eine 2022 veröffentlichte Studie zeigt, dass die konsequente Durchführung von COVID-19-Vakzinierung, das heißt insgesamt 3 Impfungen in angemessenen zeitlichen Abständen, zur Erreichung einer sehr potenten Immunität auch gegen VoCs mit Immunescape-Potenzial wie Omikron führt, die sich in der Ausbildung neutralisierender hoch avider Antikörper gegen SARS-CoV‑2 manifestierte [16]. Die Bestimmung von Surrogat-Markern zur Ermittlung des individuellen Schutzes vor Infektion oder schwerem Krankheitsverlauf erscheint deshalb nicht mehr als vordringliches Thema. Wichtiger aus heutiger Sicht ist die Vermeidung der Ausbreitung von Infektionen mit Immunescape-VoCs wie Omikron im Krankenhaus.

Die Unsicherheit, die im Frühjahr 2021 über eine optimale Strategie zur Erkennung von Infektionsketten bei Beschäftigten im Krankenhaus bestanden hat, gibt einen Hinweis darauf, dass die schnelle Erfassung von Durchbruchsinfektionen bei zukünftigen Pandemiewellen mit hoher Priorität zu behandeln ist. Auch sollten klare Handlungsempfehlungen erarbeitet und kommuniziert werden, basierend auf den jeweiligen Empfehlungen des RKI und der Ständigen Impfkommission (STIKO).

Zum Thema des Impfschutzes und der Notwendigkeit von Auffrischimpfungen bestand zum Zeitpunkt der Befragung ebenfalls keine Sicherheit und fast alle befragten Krankenhäuser äußerten großen Informationsbedarf. Dies lässt vermuten, dass eine klare und schnelle Kommunikation von Handlungsempfehlungen für weitere COVID-19-Vakzinierungen in den Krankenhäusern zu dem Zeitpunkt gefehlt hat. Dieser Befund ist von höchster Aktualität in Anbetracht einer sehr wahrscheinlichen erneuten Pandemiewelle im Herbst 2022 und der Frage nach einer Auffrischimpfung. Die Durchführung von klinischen Studien zu SARS-CoV-2-spezifischer Immunität nach weiteren COVID-19-Vakzinierungen auch in Deutschland und die Bewertung der Ergebnisse aus diesen Studien durch das RKI und die STIKO sind deshalb von großer Bedeutung.

### Stärken und Schwächen der Studie

Das Befragungsinstrument wurde in einem iterativen Verfahren mit Expert*innen aus den Bereichen Virologie, Immunologie, Infektionsmedizin, Hygiene und Public Health entwickelt und vorgetestet. Positiv zu sehen ist der hohe Rücklauf bei den Universitätskliniken sowie die Ergebnisse aus 2 unterschiedlich betroffenen Bundesländern in Deutschland. Der unterschiedlich hohe Rücklauf von Universitätskliniken und Nicht-Universitätskliniken erschwert jedoch die Vergleichbarkeit der Gruppen. Auch ist zu beachten, dass die Daten der Nicht-Universitätskliniken nicht repräsentativ für Gesamtdeutschland stehen. Es wurden aufgrund der Länge des Fragebogens keine direkten Fragen zu den Ressourcen der Kliniken gestellt, viele der gestellten Fragen gaben aber Hinweise auf diese. Die Umfrageergebnisse zeigen nur eine Momentaufnahme der sich schnell ändernden Pandemiesituation. Trotz großer Sorgfalt bei der Identifikation der richtigen Ansprechpersonen handelt es sich um eine anonyme Befragung und die Richtigkeit der Aussagen aus den jeweiligen Institutionen kann nicht überprüft werden. Durch die deskriptive Darstellung der Surveillance- und Teststrategien können keine Rückschlüsse auf deren Effizienz und Effektivität gezogen werden. Basierend auf den mit dieser Umfrage erzielten Informationen ist eine Wiederholung der Umfrage zur Analyse von Best-Practice-Anwendungen von großem Interesse.

## Fazit

Die Ergebnisse zeigen, dass alle Krankenhäuser eine schnelle Umsetzung der COVID-19-Vakzinierung ihrer Beschäftigten erreichen konnten. Eine gesicherte Belieferung mit Impfstoff kann als zentral bedeutend für eine erfolgreiche Pandemiebekämpfung gesehen werden. Auch für den Umgang mit Durchbruchsinfektionen wird von vielen Krankenhäusern eine von Expert*innen abgestimmte Surveillance-Strategie favorisiert.

## Supplementary Information




