# Supplementary material for: COVID-19 vaccination strategy for employees of German hospitals: results of a survey of infection control practitioners within the B-FAST project
Source: Bundesgesundheitsblatt Gesundheitsforschung Gesundheitsschutz. 2022 Oct 21;65(12):1299–306. [Article in German] doi: 10.1007/s00103-022-03607-x (PMC9589530; doi:10.1007/s00103-022-03607-x)
Supplement: Supplementary file 1 [file 103_2022_3607_MOESM1_ESM.pdf]

Bundesgesundheitsblatt – Gesundheitsforschung – Gesundheitsschutz, Ausgabe 12/2022

Onlinematerial zum Beitrag:

COVID-19-Impfstrategien für Beschäftigte deutscher Kliniken: Ergebnisse einer Befragung von Leitungen der (Krankenhaus-)Hygiene im Rahmen des B-FAST-Projektes

Simone Scheithauer[1*], Anna Bludau[1*], Stephanie Heinemann[2, 3], Martina Anton[4], Percy Knolle[4, 5, 6]
*geteilte Erstautorenschaft

[1] Institut für Krankenhaushygiene und Infektiologie, Universitätsmedizin Göttingen, Georg-August-Universität Göttingen, Deutschland
[2] Lokale Task Force Netzwerk Universitätsmedizin (NUM), Universitätsmedizin Göttingen, Georg-August-Universität Göttingen, Deutschland
[3] Institut für Allgemeinmedizin, Universitätsmedizin Göttingen, Georg-August-Universität Göttingen, Deutschland
[4] Institut für Molekulare Immunologie und Experimentelle Onkologie, Universitätsklinikum rechts der Isar der Technischen Universität München, Fakultät für Medizin, München, Deutschland
[5] Deutsches Zentrum für Infektionsforschung, Standort München, München, Deutschland
[6] Lokale Task Force Netzwerk Universitätsmedizin (NUM), Fakultät für Medizin der Technischen Universität München, München, Deutschland

Korrespondenzadresse:
Anna Bludau M.Sc. und Prof. Dr. Simone Scheithauer
Institut für Krankenhaushygiene und Infektiologie
Universitätsmedizin Göttingen
Robert-Koch-Straße 40
37075 Göttingen
Deutschland
krankenhaushygiene.leitung@med.uni-goettingen.de
anna.bludau@med.uni-goettingen.de

Inhalt:

Onlinematerial 1: Beschreibung der Entwicklung und Versendung des Fragebogens

Onlinematerial 2: Tabelle. Ausgewählte Charakteristika der Kliniken bezüglich ihrenr personellen, materiellen und infrastrukturellen Ressourcen

Onlinematerial 1: Beschreibung der Entwicklung und Versendung des Fragebogens

Der Fragebogen wurde in einem interdisziplinären Verfahren mit Vertreter*innen aus den Bereichen Virologie, Immunologie, Infektionsmedizin, Hygiene und Public Health entworfen. Es folgte ein Pretest mit fünf Expert*innen außerhalb der Stichprobe, um die Verständlichkeit, Relevanz und Logik der Befragung sicherzustellen. Nach der Überarbeitung des Fragebogens wurde die finale Version in das Online-Befragungstool LimeSurvey eingearbeitet. Die Funktionalität des Online-Fragebogens wurde in mehreren internen Durchläufen getestet.

Das Vorgehen zur Versendung der Einladungsschreiben unterscheidet sich zwischen den Universitätskliniken und Nicht-Universitätskliniken. Die Leitungen der (Krankenhaus-)Hygiene in den Universitätskliniken wurden über die jeweiligen Task Forces des Netzwerks Universitätsmedizin (NUM) kontaktiert und gebeten an der Befragung teilzunehmen. Zuvor wurden die Task Forces in einer Online-Veranstaltung über den Hintergrund und die Ziele der Studie sowie ihre Aufgabe als Vermittler aufgeklärt. Die Vertreter*innen der Hygiene in den Universitätskliniken erhielten mit dem Link zur Umfrage einen individuellen Zugangsschlüssel. Dieser kann nicht mit den Antworten in Verbindung gebracht werden und diente lediglich dazu, nachverfolgen zu können, welche Universitätskliniken bereits an der Befragung teilgenommen hatten. So konnte gezielt an die Teilnahme erinnert werden. Zudem konnte so sichergestellt werden, dass jede Universitätsklinik nur einmalig teilnimmt. Bei Universitätskliniken mit mehreren Standorten galt die Regel, dass die hygienebeauftragte Person des Standorts mit der höchsten Bettenanzahl stellvertretend an der Befragung teilnimmt.

Onlinematerial 2: Tabelle. Ausgewählte Charakteristika der Kliniken bezüglich ihrer personellen, materiellen und infrastrukturellen Ressourcen. Relative Angaben in Prozent. Statistische Signifikanz wurde als $p < 0,05$ definiert und signifikante Ergebnisse sind fett gedruckt. + absolute Zahlen sind gleich den relativen Zahlen.

	**Universitätskliniken** (*n* = 33)		**Nicht-Universitätskliniken** (*n* = 67)		**Gesamt** (*n* = 100)	***p*-Wert**
	relativ	absolut	relativ	absolut		
**Anzahl vollstationärer Betten**						**0,000**
Bis 99 Betten	0,0	0	7,5	5	5,0+	
100 bis 249 Betten	0,0	0	37,2	25	25,0+	
250 bis 499 Betten	0,0	0	28,4	19	19,0+	
500 bis 999 Betten	12,1	4	23,9	16	20,0+	
Ab 1.000 Betten	87,9	29	3,0	2	31,0+	
**Interne Teams**						
Interne Corona-Hotline	69,7	23	52,2	35	58,0+	0,132
Internes Probeentnahme Team	63,6	21	71,6	48	69,0+	0,492
Internes Contact-Tracing-Team	84,8	28	52,2	35	63,0+	**0,002**
**Zusätzlich eingestelltes Personal für Einlasskontrolle**	54,5	18	35,9	24	42,0+	**0,049**
**Vor-Ort-Diagnostik von mikrobiologisch-virologischen Untersuchungsproben**	90,9	30	41,8	28	58,0+	**0,000**
